# Supplementary material for: Nocturnal increase in cerebrospinal fluid secretion as a circadian regulator of intracranial pressure
Source: Fluids Barriers CNS. 2023 Jun 23;20:49. doi: 10.1186/s12987-023-00451-2 (PMC10290349; doi:10.1186/s12987-023-00451-2)
Supplement: Supplementary file 1 — Additional file 1: Figure S1. Cerebral perfusion pressure. Figure S2. 86Rb+ uptake in excised rat lateral choroid plexus as a function of time. Figure S3. Correlation analysis of the nightly change in paired intracranial pressure and mean arterial pressure in patients and freely behaving rats. Figure S4. NKCC1 and Na+/K+-ATPase protein expression in choroid plexus acutely excised in the light and the dark phase. Figure S5. Quantification of brain water content in brains excised during the lightand the darkphase. [file 12987_2023_451_MOESM1_ESM.pdf]

ADDITIONAL FILE 1

Supplementary figure 1
Cerebral perfusion pressure (CPP) acquired by deducting the ICP from the MAP in the patient group in both the dark phase and the light phase, n = 21. ns; not significant.

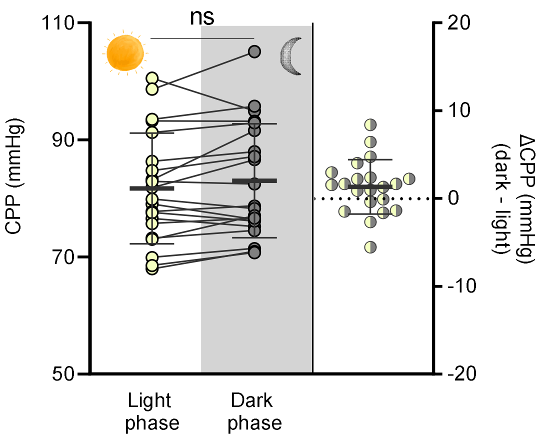

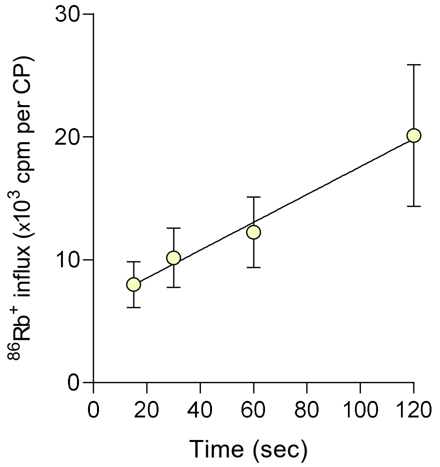

Supplementary figure 2.
$^{86}Rb^{+}$ uptake in excised rat lateral choroid plexus as a function of time of incubation illustrating time-linearity from 15 sec to 120 sec, n = 7 choroid plexuses (CP) at each time point divided over 3 individual experiments. The $^{86}Rb^{+}$ uptake as a function of time does not intercept the zero time point, which we suspect is due to 'background' $^{86}Rb^{+}$ trapped in the tissue despite the washing procedure. This extracellular $^{86}Rb^{+}$ is partially corrected for by the extracellular marker ^{3}H-mannitol (see Methods), which however diffuses slower than the much smaller Rb^{+}, potentially leaving the extra background $^{86}Rb^{+}$ counts, which, however, does not influence the time linearity from 15-120 sec, but merely cause the parallel shift.

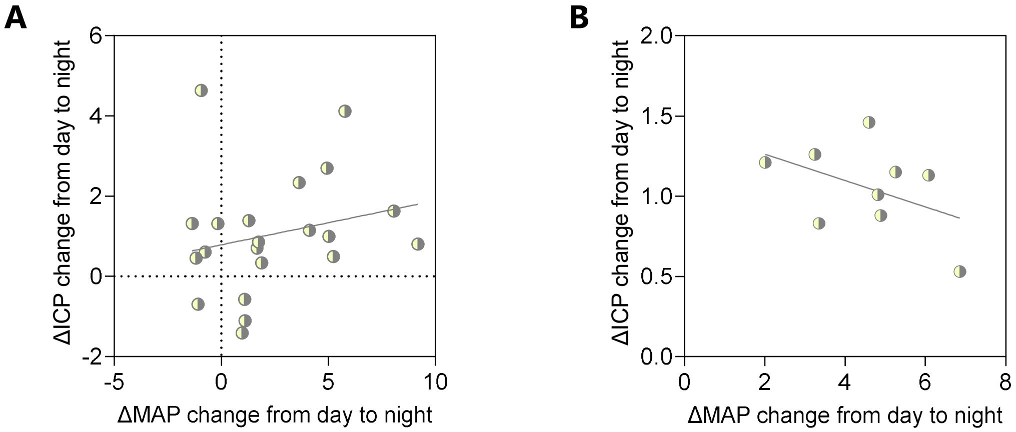

Supplementary figure 3.
Correlation analysis of the nightly change in paired intracranial pressure (ICP) and mean arterial pressure (MAP) from **A,** patient cohort (n = 21, r^2 = 0.05, P = 0.33, Spearman correlation) and **B,** freely behaving rats (n = 6, r^2 = 0.06, P = 0.23, Pearson correlation).

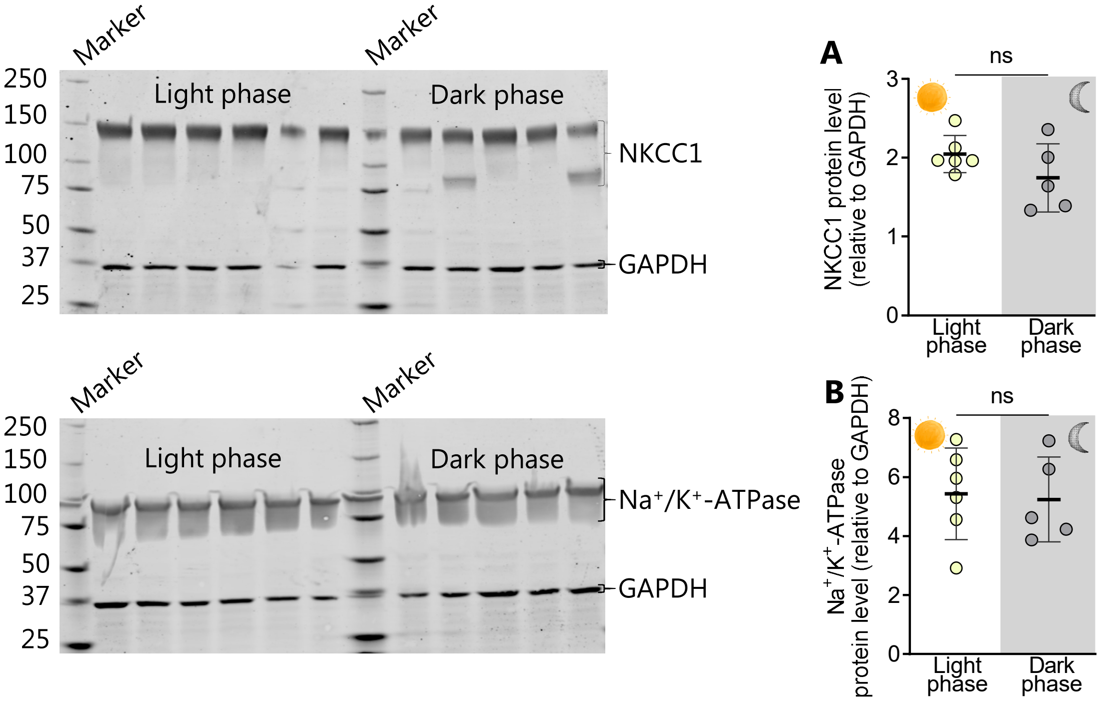

Supplementary figure 4. Na^+,K^+,$2Cl^-$ cotransporter (NKCC1), and Na^+/K^+-ATPase protein expression in choroid plexus acutely excised in the light and the dark phase. Analyzed by SDS-PAGE (Mini-protean TGX, precast 4-20%, Bio-Rad) and immunoblotting (Immobilon-FL membranes, Millipore, MA), left panel, utilizing glyceraldehyde 3-phosphate dehydrogenase (GAPDH) as a loading control. Primary antibodies included sheep anti-NKCC1, 1:400 (SO22D, Dundee, anti-sheep), mouse anti-Na^+/K^+-ATPase-$\alpha1$,1:60 (a6F, DSHB), chicken anti-GAPDH, 1:800 (AB2302, Millipore) and detection performed using fluorophore-conjugated secondary antibodies (LI-COR) and scanned on an Odyssey CLx imaging system. No significant difference was observed between the two phases for **A,** NKCC1 (dark phase, 1.7 ± 0.4, n = 5, vs light phase, 2.0 ± 0.2, n = 6, P = 0.18), or **B,** Na^+/K^+-ATPase α1 (dark phase, 5.2 ± 1.4, n = 5, vs light phase, 5.4 ± 1.6, n = 6, P = 0.84). Data are presented as mean ± SD, and statistical significance (P < 0.05) determined using unpaired t-test. ns; not significant.

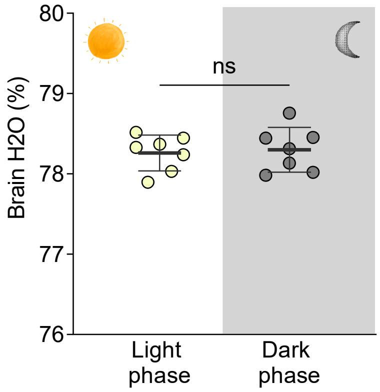

Supplementary figure 5. Quantification of brain water content in brains excised during the light (ZT 8h) and the dark (ZT 20h) phase. Swiftly excised rat brains were weighed, dried at 100°C for a minimum of 72 h before re-weighing. Brain water content in percentage did not differ between phases (dark phase 78.3 ± 0.3 % vs light phase 78.3 ± 0.2 %, n = 7, P = 0.78). Data presented as mean ± SD and statistical significance determined using unpaired t-test. ns; not significant.
